# The reproducibility of electronic color measurements of the marginal gingiva

**DOI:** 10.1007/s00784-020-03345-x

**Published:** 2020-06-22

**Authors:** Henning Staedt, Eva Mally, Herbert Scheller, Stefan Wentaschek, Peer Wolfgang Kämmerer, Adrian Kasaj, Alessandro Devigus, Karl Martin Lehmann

**Affiliations:** 1 Department of Prosthodontics and Materials Science, University Medical Centre Rostock, Strempelstraße 13, 18057 Rostock, Germany; 2 Department of Prosthodontics and Materials Science, University Medical Centre Mainz, Augustusplatz 2, 55131 Mainz, Germany; 3 Department of Oral and Maxillofacial Surgery, Plastic Surgery, University Medical Centre Mainz, Mainz, Germany; 4 Department of Conservative Dentistry and Periodontology, University Medical Centre Mainz, Mainz, Germany; 5 Bülach, Switzerland

**Keywords:** Color, Electronic color measurement, Class V, Esthetic dentistry, Gingiva, Soft tissue, Periodontal, Easyshade

## Abstract

**Introduction:**

This study evaluated the reproducibility of electronic color determination system evaluations of the marginal gingiva, which could be important for adhesive cervical fillings or prosthetic restorations that imitate the gingiva.

**Material and methods:**

In 50 subjects, the L*, a*, and b* color coordinates were evaluated five times at a point in the marginal area of a central incisor using different electronic color determination systems: (SP) Shadepilot, (ES) Easyshade, (CE) Crystaleye, and (SV) X-Rite. The mean color difference (ΔE) and its standard deviation between the five measurements from each participant were calculated separately for each device. Further ICC for interdevice reliability was determined.

**Results:**

The L*, a*, and b* color coordinates and ΔE values differed significantly among the systems (*p* < 0.001). Within each patient and measurement system, ΔE ranged from 1.4 to 3.2 (SD 1.1–2.5), L* from 2.6 to 5.7 (SD 2.6–5.7), a* from 11.9 to 21.3 (SD 3.6–3.9), and b* from 15.1 to 28.9 (SD 1.7–4.3). Interdevice reliability ranged between 0.675 and 0.807.

**Conclusions:**

Color determination of the marginal gingiva using the electronic tooth color determination systems tested herein showed limited reproducibility. The results obtained with the different measurement systems differed enormously.

**Clinical relevance:**

These results show that the electronic color measurement devices tested allow no high reproducible determination of color coordinates of the marginal gingiva.

## Introduction

As society ages and more people retain their natural teeth, the incidence of anterior restorations continues to increase, particularly cervical fillings; thus, Class V restorations are increasingly necessary for oral rehabilitation [1, 2]. Class V restorations are applied in areas of occlusal dysfunction and abrasive processes, where the fillings must withstand shrinkage of the resin material and bonding and deformation during static and dynamic occlusal dysfunction [3, 4]. Composite restorations created in this context often require esthetic color adjustment [5]. Typically, color matching refers to the color of the affected tooth. However, in certain situations, to avoid surgical treatment [6], it may be beneficial to use gingiva-colored composites or ceramic materials to achieve better esthetic results and the ideal proportions of red and white [7, 8]. Consequently, color matching of the marginal gingiva is necessary and it is a challenge to achieve color harmonization for natural-looking teeth.

Traditionally, to match tooth or gingival color, the dentist referred to color keys to identify the target color [9]. In the last decade, however, color-measuring devices have been used to overcome the disadvantages of visual color matching, which include observer factors like color blindness, fatigue, light conditions, and health impairments. These color-measurement devices have the potential to detect colors more precisely and reproducibly, by determining the color coordinates and dental color shades and calculating the color differences (ΔE) between objects [10–18]. The ΔE values are calculated using formulas that enable quantitative evaluation of ΔE. The most frequently used formula is derived from the CIE L*a*b* system. Several studies have reported ΔE thresholds that are clinical acceptable and result in perceptible ΔE [19]. Indeed, 50% of examiners are able to perceive a ΔE value < 1 when observing opal monochromatic samples under controlled conditions. Therefore, the minimum ΔE for discriminating a mismatch between the composite and tooth color in the oral cavity varies from study to study [20]. Although electronic shade-measuring systems are used for color determination, few studies have evaluated the reproducibility of electronic gingival color determination, particularly in the context of adhesive restorations of Class V defects [2, 21, 22].

**Fig. 1:**
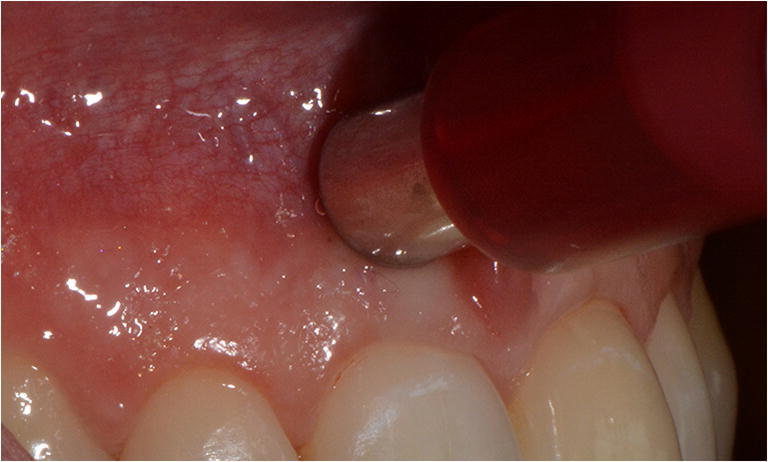
Color measurement of the marginal gingiva with the ES device

Therefore, this study evaluated the reproducibility of electronic color-determination system evaluations of the marginal gingiva (Figs. 1).

**Fig 3:**
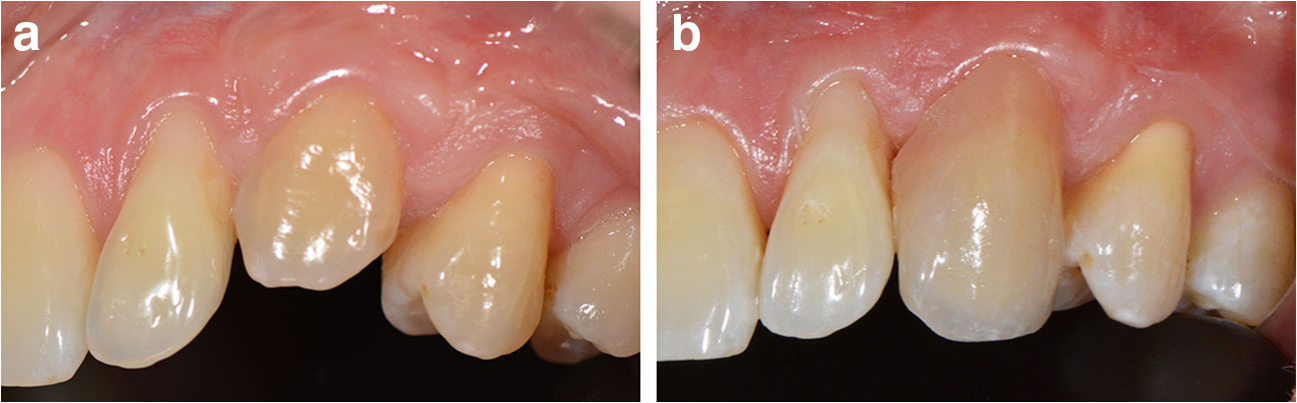
Clinical situation before (**a**) and after (**b**) a prepared ceramic restoration with gingiva colored parts

## Materials and methods

### Color-measuring devices

**Fig. 2:**
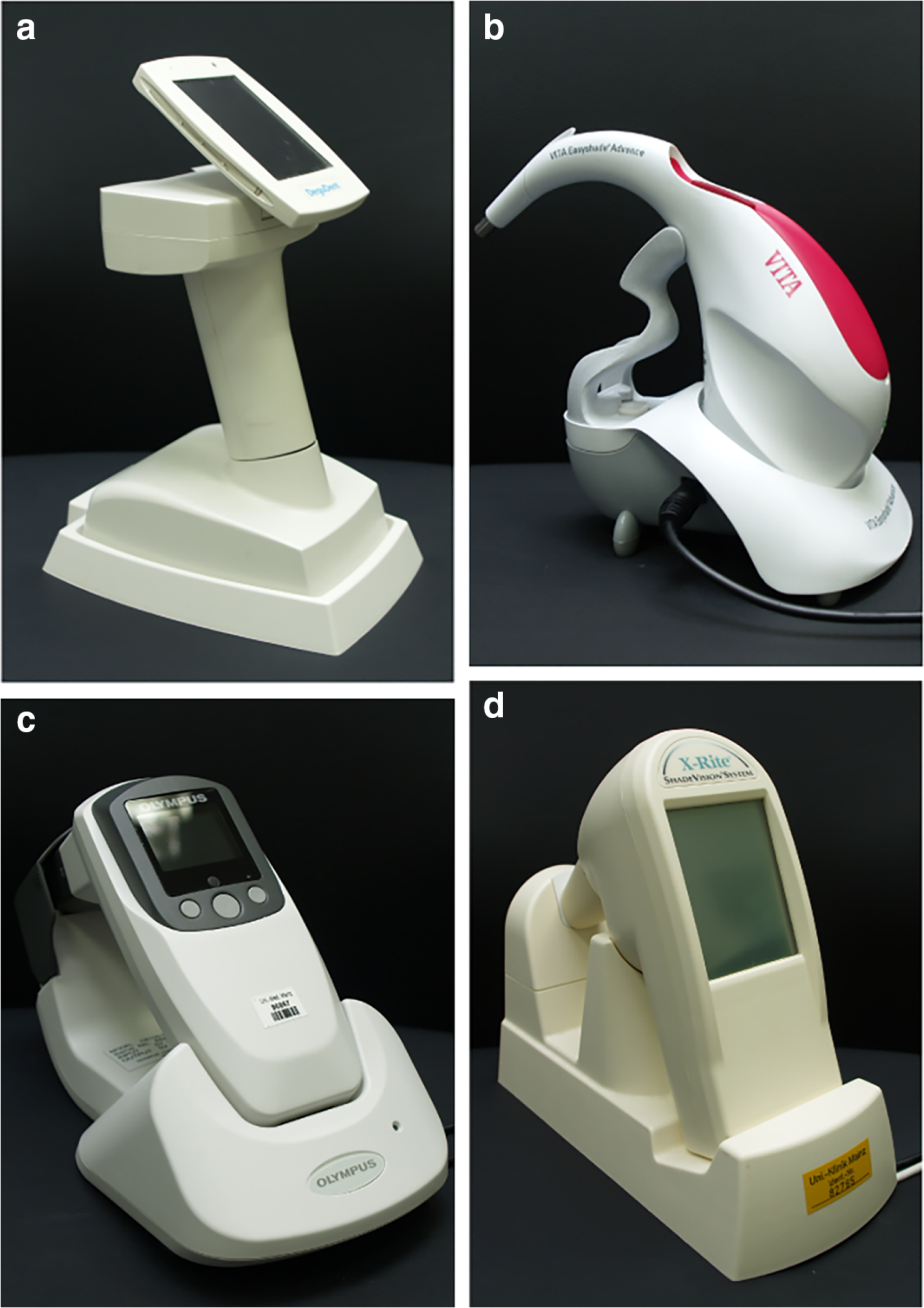
Color measurement devices used for gingival color measurements SP (**a**), ES (**b**), CE (**c**), and SV (**d**)

This study examined four commercial dental color-measuring devices (Table 1) with different operating modes: Shadepilot (SP) (Figs. 2a, Hanau, Germany) the Easyshade Advance (ES) (Fig. 4; VITA Zahnfabrik H. Rauter GmbH & Co. KG, Bad Säckingen, Germany); (Figs. 2c, (CE) (Fig. 5) Olympus America, Center Valley, PA, USA); and X-Rite ShadeVision (SV) ((Figs. 2d) X-Rite, Grand Rapids, MI, USA) [13, 15]. Table 1 summarizes the specifications of the devices. Each device was used according to the manufacturer’s instructions and this investigation was performed in terms of quality assurance. Of the four devices, the SP, ES, and CE are spectrophotometric devices and the SV is a colorimetric device; all have different operating modes.

**Table 1:** Technical specifications of the electronic measurement devices studied

Device	Contact mode	Data acquisition	Light source	Wavelength	Spectral resolution	First marketed
SP	Non-contact	Spectrophotometric	LED	400–720 nm	10 nm	2006
ES	Contact	Spectrophotometric	LED	400–700 nm	25 nm	2011
CE	Non-contact	Spectrophotometric	LED	400–720 nm	10 nm	2008
SV	Non-contact	Colorimetric	Filament light	N/A	N/A	2001

All measurements were performed under the same standardized test conditions by one trained operator (five measurements per tooth) during restorative and prosthetic restorations (Fig. 3a, b)

The electronic device SP was calibrated using the white and green calibration tiles provided by the manufacturer before obtaining images of teeth. The optical hand piece was held at 90° to the target tooth and flush against the gingival matrix. The SP device measured the CIE L*a*b* values for each tooth and automatically selected the closed shade tab from the internal database of the manufacturer. Tooth analysis mode was chosen and the color coordinates of the marginal area of the tooth 11 were recorded by manual selection. The ES system was calibrated using the white calibration standard as per the manufacturer’s instructions, with the hand piece in its stand. Tooth area analysis mode was used and only the color information of a point of the marginal area of the tooth 11 were analyzed. The CE system was calibrated using the calibration plate on the docking station before measurements and the device was positioned to capture the tooth image. Spectral data for the cervical, central, and incisal thirds of the tooth were analyzed. The best shade match obtained with the VC-system, and CIE L*a*b* values, was calculated for a point of the marginal gingiva of the tooth 11. The SV device, calibrated on the docking station before and between tooth measurements, measured the selected point of the marginal gingiva of the tooth 11.

### Patients

This study enrolled 50 patients (34 females, 16 males, age 22-29 years (mean age: 24.8 years), which received. During measurements, they were instructed to place their heads against the headrest of a dental chair and relax. The color measurement devices were used and calibrated according to the manufacturers’ instructions. The CIE L*a*b* values were recorded five times for each patient without removal of the instruments. The region of interest was a point of the gingiva with a distance of 2 mm to the gingival margin considering the tooth axis of the tooth 11. Patients with abnormal discoloration were excluded. All patients underwent a professional tooth cleaning before the examination.

### Test conditions

All measurements were made by a single trained operator under standardized test conditions to avoid external influences. The operator was trained to keep the measuring points during the measurements. A repositioning jig was not used. The illumination source (Just Normlicht, Weilheim an der Teck, Germany) was set at 6,500 K and 1,000 Lux. Natural daylight was excluded using an opaque optical louver. The shade of slightly moistened gingiva was examined and the patient’s clothing was covered with a cloth to minimize visual interference.

### Statistical analyses

The data were imported into SPSS ver. 19.0 (SPSS, Chicago, IL, USA) or SAS ver. 9.2 (SAS Institute, Cary, NC, USA). Reproducibility was assessed by calculating the mean and standard deviation (SD) of the color coordinates (L*, a*, and b*) with each color measuring system. The color distances (Δ*E*) among the measurements were calculated for each patient and each device using the following equation:

$$ \Delta E={\left[{\left(L\ast \mathrm{i}\hbox{--} L{\ast}_{\mathrm{i}\mathrm{i}}\right)}^2+{\left(a{\ast}_{\mathrm{i}}\hbox{--} a{\ast}_{\mathrm{i}\mathrm{i}}\right)}^2+{\left(b{\ast}_{\mathrm{i}}\hbox{--} b{\ast}_{\mathrm{i}\mathrm{i}}\right)}^2\right]}^{\frac{1}{2}} $$

where i and ii are two different measurements [18]. The color coordinates of the color-measuring devices were compared using analysis of variance (ANOVA) with the Bonferroni adjustment to control for multiple testing. *P* values < 0.016 were deemed to indicate significance. Further intraclass correlation coefficients (ICC) for the color coordinates (L*, a*, and b*) and for the color distances (Δ*E*) were calculated to determine interdevice reliability.

## Results

It was shown both in the L*, a*, and b* color coordinates and in the calculated color differences Δ*E* different results with wide ranges, so L* color coordinates ranged from 49 to 78.7, a* coordinates from 3.6 to 31.2, b* coordinates from 8.6 to 45.6, and the calculated color differences Δ*E* for each device from 0.1 to 19.1. Table 2 shows the mean, standard deviations, minimum, and maximum values of L*, a*, and b* coordinates and calculated color differences Δ*E* for each color measurement device.

**Table 2:** Mean, standard deviation (sd), minimum (min), maximum (max) of the L*, a*, and b* color coordinates with the calculated color difference (∆*E*) between the measurements for each patient and for each color measurement device (SP, Shadepilot; ES, Easyshade; CE, Chrystaleye; SV, Shadevision)

		*n*	Mean	sd	min	max
L*	SP	250	56.3	2.6	50.1	62.6
	ES	250	68.5	5.7	49	78.7
	CE	250	56.0	3.3	49.3	67.2
	SV	250	59.4	2.9	52.2	67.5
a*	SP	250	23.1	3.5	14.6	31.2
	ES	250	11.9	3.9	3.6	24.6
	CE	250	15.0	3.7	4.4	23.4
	SV	250	21.3	3.6	10.4	30.2
b*	SP	250	19.9	2.1	14.7	29.3
	ES	250	28.9	4.3	19.3	45.6
	CE	250	15.1	1.9	8.6	20.5
	SV	250	15.8	1.7	11.0	20.8
∆*E*	SP	500	1.5	1.2	0.1	9.2
	ES	500	3.2	2.5	0.2	19.1
	CE	500	1.4	1.1	0.1	9.3
	SV	500	2.6	1.6	0.2	10.1

Only the L* coordinates and the Δ*E* values between the system A and C systems differed not significantly (*p* > 0.016)

Table 3 shows the intraclass correlation coefficients (ICC) for interdevice reliability for the L*, a*, and b* color coordinates and color distances Δ*E* for the devices tested. These ICC ranged from 0.675 to 0.807.

**Table 3:** Intraclass correlation coefficients for interdevice reliability for L*, a*, and b* color coordinates and color distances Δ*E* (SP, Shadepilot; ES, Easyshade; CE, Chrystaleye; SV, Shadevision)

	L*SP	L*ES	L*CE	L*SV	a*SP	a*ES	a*CE	a*SV	b*SP	b*ES	b*CE	b*SV	∆*E* *SP	∆*E* *ES	∆*E* *CE	∆*E* *SV
L*SP	-	0.749	0.745	0.745												
L*ES	0.749	-	0.750	0.748												
L*CE	0.745	0.750	-	0.745												
L*SV	0.745	0.748	0.745	-												
a*SP					-	0.787	0.766	0.755								
a*ES					0.787	-	0.781	0.786								
a*CE					0.766	0.781	-	0.765								
a*SV					0.755	0.786	0.765	-								
b*SP									-	0.777	0.757	0.755				
b*ES									0.777	-	0.807	0.803				
b*CE									0.757	0.807	-	0.749				
b*SV									0.755	0.803	0.749	-				
∆*E* *SP													-	0.781	0.675	0.721
∆*E* *ES													0.781	-	0.795	0.705
∆*E* *CE													0.675	0.795	-	0.731
∆*E* *SV													0.721	0.705	0.731	-

## Discussion

Pink composite or ceramic restorations are a viable option for restoring Class V defects or perform optimal red esthetics with prosthetic restorations [8]. Even with challenging restorations, such as massive recession defects (Miller class II or above) or pigmentation of the marginal gingiva, composite restorations are an esthetic alternative to invasive surgical procedures for treating recession [6, 23]. However, visual evaluation of the shade of the gingiva has disadvantages because it is affected by light conditions (i.e., metamerism) or dyschromatopsia. Shade guides and electronic measurement systems are available for evaluating the shade of natural teeth. Several shade guides have been developed for different requirements, such as color matching in prosthetic restorations or detecting color changes during a bleaching process. However, only a few shade guide systems are available for evaluating the shade of gingival regions, and these are subject to coverage errors [9]. Ghinea et al. declared that there is no optimal gingival shade guide [24].

Therefore, a more objective and reproducible method for evaluating gingival color would be helpful. While electronic shade-taking systems are generally used for reproducible determination of tooth color [10, 13, 15, 16], these systems may also have the potential to evaluate gingival color reproducibly. The color coordinates of the gingiva vary widely and differ between females and males [25]. It is not clear whether tooth color measurement systems can record the strongly scattered color information of the marginal gingiva, especially because one of the systems tested (device ES) generates color information from reflected light. It is also not clear to what extent the thickness and type of gingiva, the region within the oral cavity [26], and the contact mode of the ES device affect color measurements. In fact and a strength of this study is that it was shown that the color measurement systems tested generated different color coordinates of gingival tissue that is shown by the low ICCs. In contrast to this, a limitation of this study is that no repositioning jig was used. Although such a jig was not used, which could have caused an extra variance of the results, this is not surprising because although dental color measuring systems have high reproducibility, they are not CIE-compliant. It was first demonstrated a difference between these systems and a CIE-compliant system in 2010 [15]. The SDs of the L*, a*, and b* color coordinates ranged from 1.6 to 5.7, and the calculated ΔE for each proband and measuring system also differed, as expected but first shown in this study. Several formulas are available for calculating Δ*E* [27] and the Δ*E* of each system has a SD visible to humans [19]. Therefore, the small differences in color of the gingival regions detected could also result from the measurement uncertainty of the systems. This is not surprising given that the instruments were designed for determining the shade of natural teeth, which have different color coordinates. Particularly, contact measurement systems, such as the ES system, could affect tissue perfusion during gingival color measurements, resulting in color changes that is a further limitation when interpreting the results. Therefore, soft tissue colors should be measured with non-contact systems. Considering the inherent uncertainties of gingiva measurements, the SP and CE systems showed the highest reliability for gingival color measurements of the devices tested. However, all these devices allow no high reproducible determination of color coordinates of the marginal gingiva.

## Conclusion

The electronic tooth color determination systems evaluated herein are limited in terms of the reproducibility of evaluations of marginal gingival color. Furthermore, the results of the different measurement systems differ enormously; this should be considered in future clinical studies, whereas the systems SP and CE showed a higher reproducibility compared with the other two systems tested in this study. However, all systems tested in this study are not suitable for high reproducible determination of color coordinates of the marginal gingiva.
